# Diagnosis, Management, and Follow-Up of Human Papillomavirus Type 16 (HPV-16) Lesions in the Oral Cavity

**DOI:** 10.7759/cureus.109263

**Published:** 2026-05-20

**Authors:** Mita Patel, Antoine Bruneau, François Le Pelletier de Glatigny, Ihsene Taihi

**Affiliations:** 1 Oral Surgery, Sorbonne University, Paris, FRA; 2 Oral Surgery, Pitié-Salpêtrière University Hospital, Assistance Publique-Hôpitaux de Paris (AP-HP), Sorbonne University, Paris, FRA; 3 Pathology (Anatomy and Cytology), Pitié-Salpêtrière University Hospital, Assistance Publique-Hôpitaux de Paris (AP-HP), Sorbonne University, Paris, FRA; 4 Oral Surgery, Rothschild Hospital, Assistance Publique-Hôpitaux de Paris (AP-HP), Sorbonne University, Paris, FRA

**Keywords:** biopsy, human papillomavirus 16, mouth mucosa, oral leukoplakia, squamous cell carcinoma

## Abstract

Human papillomavirus (HPV), particularly genotype HPV-16, is strongly associated with oropharyngeal carcinogenesis, although its role in oral cavity lesions remains uncertain. We report the case of a 42-year-old man presenting with leukoplakic lesions affecting the vermilion borders of both lips. Histopathological examination showed non-dysplastic hyperkeratosis, with koilocytosis suggestive of viral infection, and HPV-16 was confirmed by polymerase chain reaction (PCR) analysis. Early recurrence occurred shortly after surgical excision, leading to the implementation of regular clinical surveillance. At the two-year follow-up, no recurrence or malignant transformation was observed, and the lesions remained in complete clinical remission. This case highlights the diagnostic and therapeutic challenges posed by HPV-associated leukoplakic lesions of the oral cavity and emphasizes the importance of careful long-term monitoring.

## Introduction

Human papillomavirus (HPV) infection is recognized as a major etiological factor in several epithelial malignancies. Viral carcinogenesis is primarily driven by the integration of the viral genome into host cell DNA, leading to overexpression of the viral oncoproteins E6 and E7, which inactivate tumor suppressor proteins such as p53 and pRb and promote genomic instability [1-5]. High-risk genotypes, particularly HPV-16 and HPV-18, are strongly associated with malignant transformation [5,6].

HPV is implicated in approximately 39% of vulvar carcinomas, 90% of anal carcinomas, and between 6% and 25% of head and neck squamous cell carcinomas, with a particularly high prevalence in oropharyngeal squamous cell carcinoma [7,8]. While the causal relationship between HPV-16 infection and oropharyngeal cancer is well established, its role in oral cavity carcinogenesis remains controversial, with highly variable prevalence rates reported in the literature [5,9-11]. In the oral cavity, leukoplakic and verrucous lesions may have multiple etiologies, and the differential diagnosis includes frictional keratosis, oral lichen planus, chronic hyperplastic candidiasis, verruca vulgaris, and early squamous cell carcinoma.

More than 200 HPV genotypes have been identified, differing in their epithelial tropism and oncogenic potential [6]. HPV-16 is the most prevalent high-risk genotype worldwide and has increasingly been detected in the oral cavity, possibly reflecting changes in sexual practices and oro-genital transmission routes [8]. Oral HPV infection prevalence has been estimated at approximately 7% in the general population, with higher rates observed in males than in females and a peak prevalence in middle-aged individuals. It has also been associated with high-risk sexual behaviors, including multiple sexual partners and oral sexual practices [8,12]. However, the prognostic significance of HPV detection in oral lesions remains unclear, particularly in the context of potentially malignant oral cavity disorders.

Within the oral cavity, oral potentially malignant disorders (OPMD) are defined by the World Health Organization (WHO) as mucosal abnormalities associated with an increased risk of developing oral cancer [1]. Among these conditions, leukoplakia represents the most common clinical entity, with an estimated malignant transformation rate of approximately 10% [2]. The risk of transformation is primarily related to the degree of epithelial dysplasia; however, additional cofactors, including tobacco use, alcohol consumption, and possibly viral infections such as HPV, may influence lesion behavior [3,4,9].

The presence of HPV-16-positive leukoplakic lesions in the oral cavity, therefore, represents a clinical and therapeutic challenge. Clinicians must determine whether early intervention is warranted or whether careful surveillance is sufficient. In this context, we report a rare case of HPV-16-associated leukoplakia of the vermilion border, characterized by early recurrence and favorable long-term follow-up.

## Case presentation

A 42-year-old male was referred to the Oral Surgery Department of Rothschild Hospital for evaluation of asymptomatic lip lesions. The patient had no significant medical or surgical history. He reported occasional alcohol consumption and a 10-pack-year smoking history. No history of HPV vaccination was reported. 

The lesion on the upper lip had appeared 30 months earlier, and the lower lip lesion had developed nine months prior to consultation. Both lesions showed fluctuating progression, with episodes of regression and recurrence.

**Figure 1:**
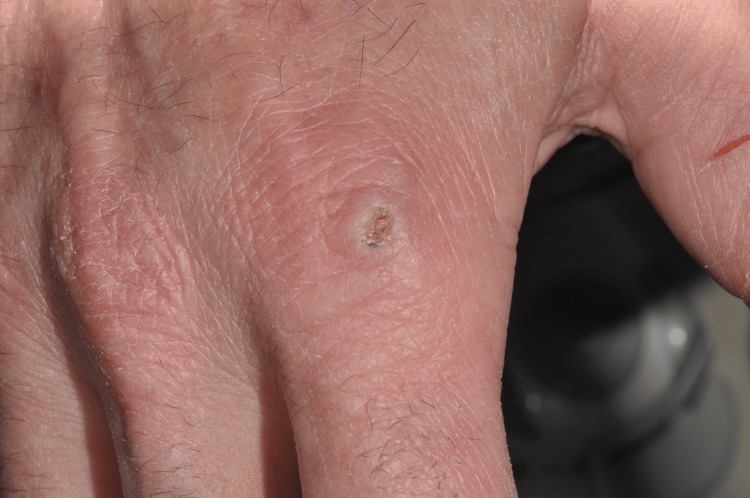
Verrucous skin lesions on the right index finger

**Figure 2:**
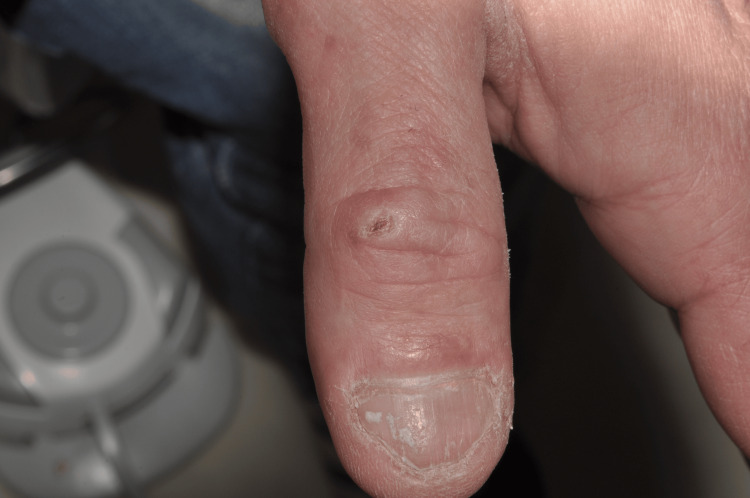
Verrucous skin lesions on the left thumb

**Figure 3:**
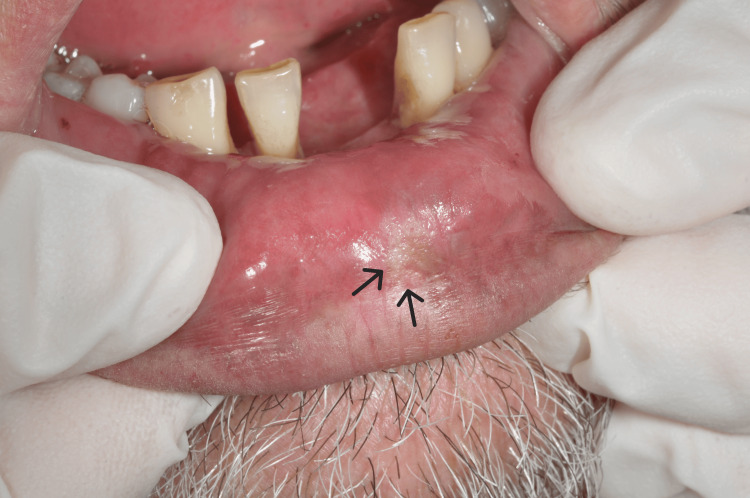
Leukoplakic area on the vermilion borders of the lower lip, measuring over one centimeter, with a yellowish appearance, well-defined edges, and a homogeneous surface (arrows)

**Figure 4:**
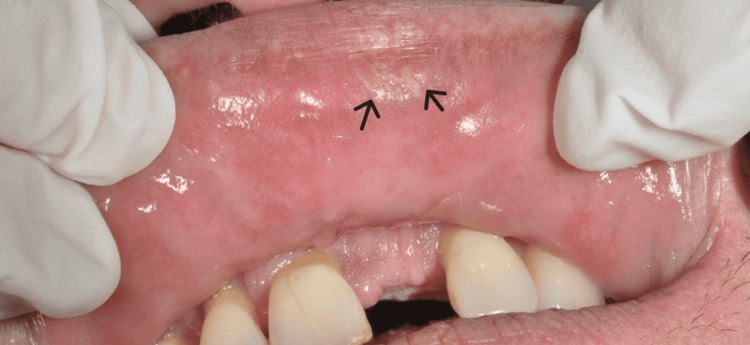
Leukoplakic area on the vermilion border of the upper lip, measuring over one centimeter, with a yellowish appearance, well-defined edges, and a homogeneous surface (arrows)

Clinical examination revealed two verrucous skin lesions on the right index finger (Figure 1) and left thumb (Figure 2). Intraoral examination demonstrated two leukoplakic lesions on the vermilion borders of the lower (Figure 3) and upper lips (Figure 4), each larger than one centimeter, with well-defined margins and homogeneous yellowish surfaces (Figure 3). No lymphadenopathy or systemic symptoms were observed.

**Figure 5:**
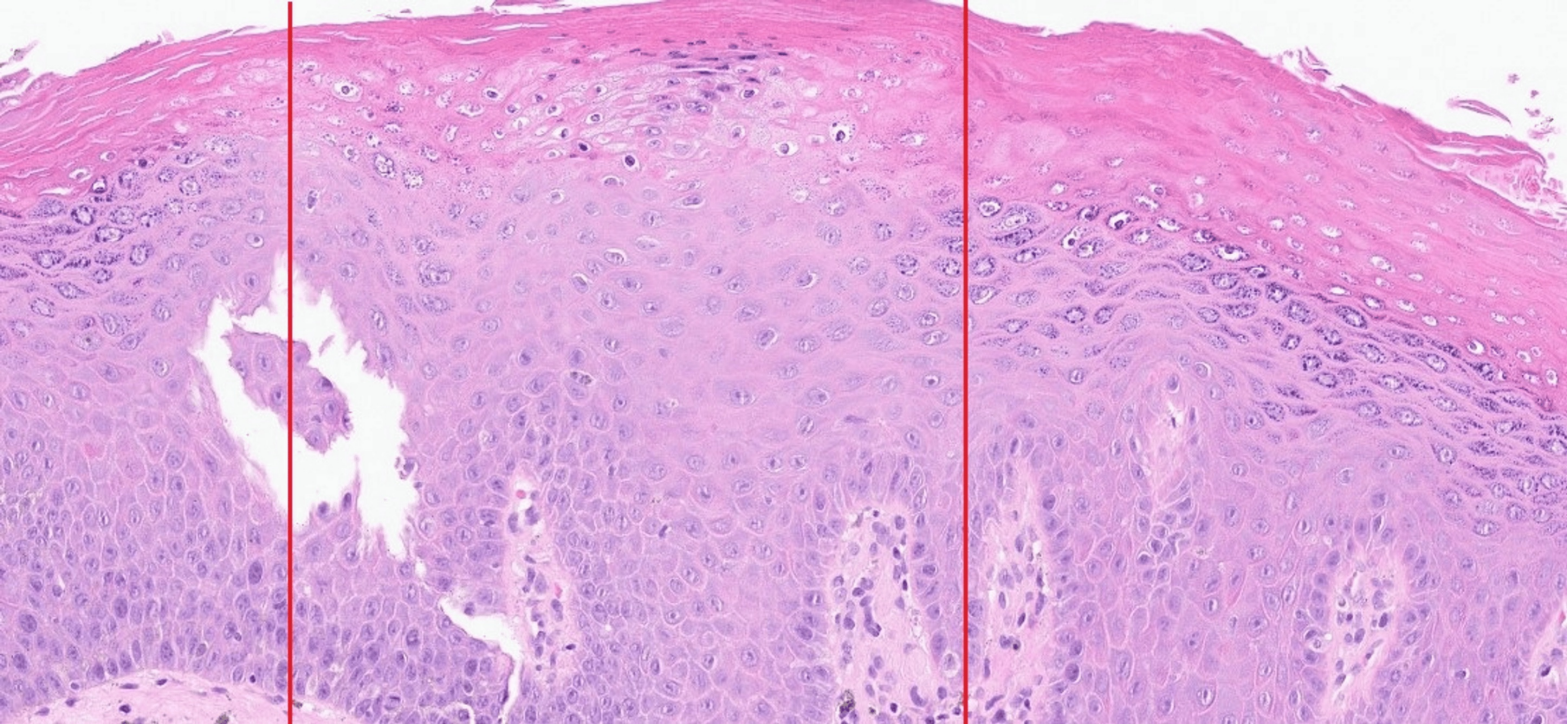
Histological section showing central parakeratosis with orthokeratosis on the right and left sides

**Figure 6:**
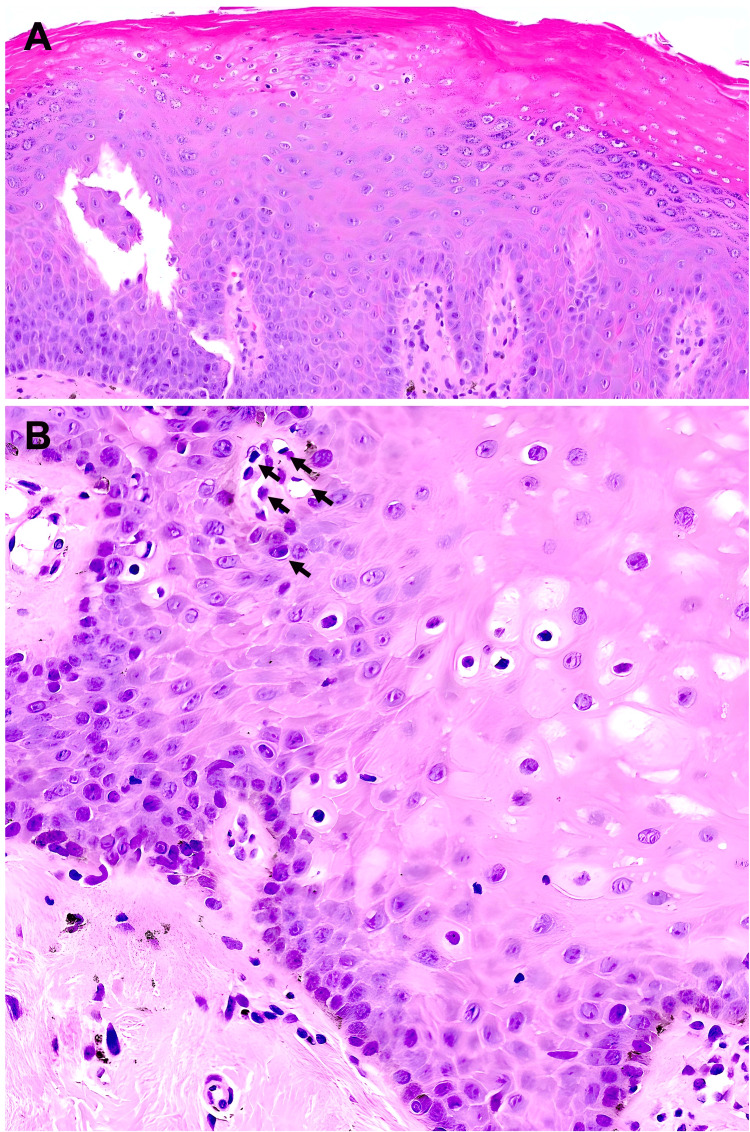
Histological findings A: histological section showing central parakeratosis with orthokeratosis on the right and left sides; B: histological section showing hyperplasia without dysplasia, with the presence of koilocytes (arrows).

Given the small size (approximately 1 cm) and well-circumscribed clinical appearance of the lesions, an excisional biopsy was performed for both diagnostic and therapeutic purposes. The absence of clinical features suggestive of malignancy supported a conservative surgical approach without safety margins. An incisional biopsy was not performed, as complete excision was feasible and allowed for both definitive diagnosis and initial management. Histopathological examination revealed non-dysplastic hyperkeratosis consistent with leukoplakia (Figure 5). The epithelium showed acanthosis with increased epithelial thickness and surface hyperkeratosis. Basal cell morphology was preserved, with no architectural or cytological features of dysplasia. A mild chronic inflammatory infiltrate was observed in the underlying connective tissue. HPV-related cytopathic changes were identified, including koilocytosis defined by perinuclear clearing, nuclear enlargement, and irregular nuclear contours (Figure 6). These findings were considered suggestive of HPV infection rather than reactive epithelial changes, particularly in the context of positive HPV-16 polymerase chain reaction (PCR) testing. HPV detection was performed using PCR targeting viral DNA, followed by specific genotyping for high-risk HPV types, which confirmed HPV-16 positivity. Histopathological differential diagnoses included frictional keratosis, verruca vulgaris, and chronic hyperplastic candidiasis; however, the presence of koilocytosis and molecular confirmation supported a diagnosis of an HPV-associated lesion. The final clinicopathological diagnosis was HPV-16-associated leukoplakia of the vermilion border. 

An early clinical reappearance of the lesions was observed one week postoperatively. The lesions presented as well-demarcated, whitish plaques located on the same vermilion areas, with a similar clinical appearance to the initial presentation. Given the very short interval following surgery, this finding was considered more consistent with incomplete excision or persistent epithelial alteration rather than true biological recurrence. No repeat biopsy was performed at that stage, as the lesions showed no clinical features suggestive of malignancy and the initial histopathological examination had confirmed the absence of dysplasia.

Following multidisciplinary discussion with head and neck oncology specialists, a conservative management strategy was adopted, consisting of regular clinical surveillance every four months. The patient was advised regarding smoking cessation and the reduction of alcohol consumption. He had no history of HPV vaccination. The cutaneous verrucous lesions on the fingers were also monitored and were considered a possible source of viral persistence or reinoculation. Alternative treatments such as cryotherapy, laser ablation, electrocoagulation, and photodynamic therapy may be considered for persistent or recurrent lesions. In this case, these options were not pursued due to the absence of dysplasia and the favorable clinical evolution under surveillance.

**Figure 7:**
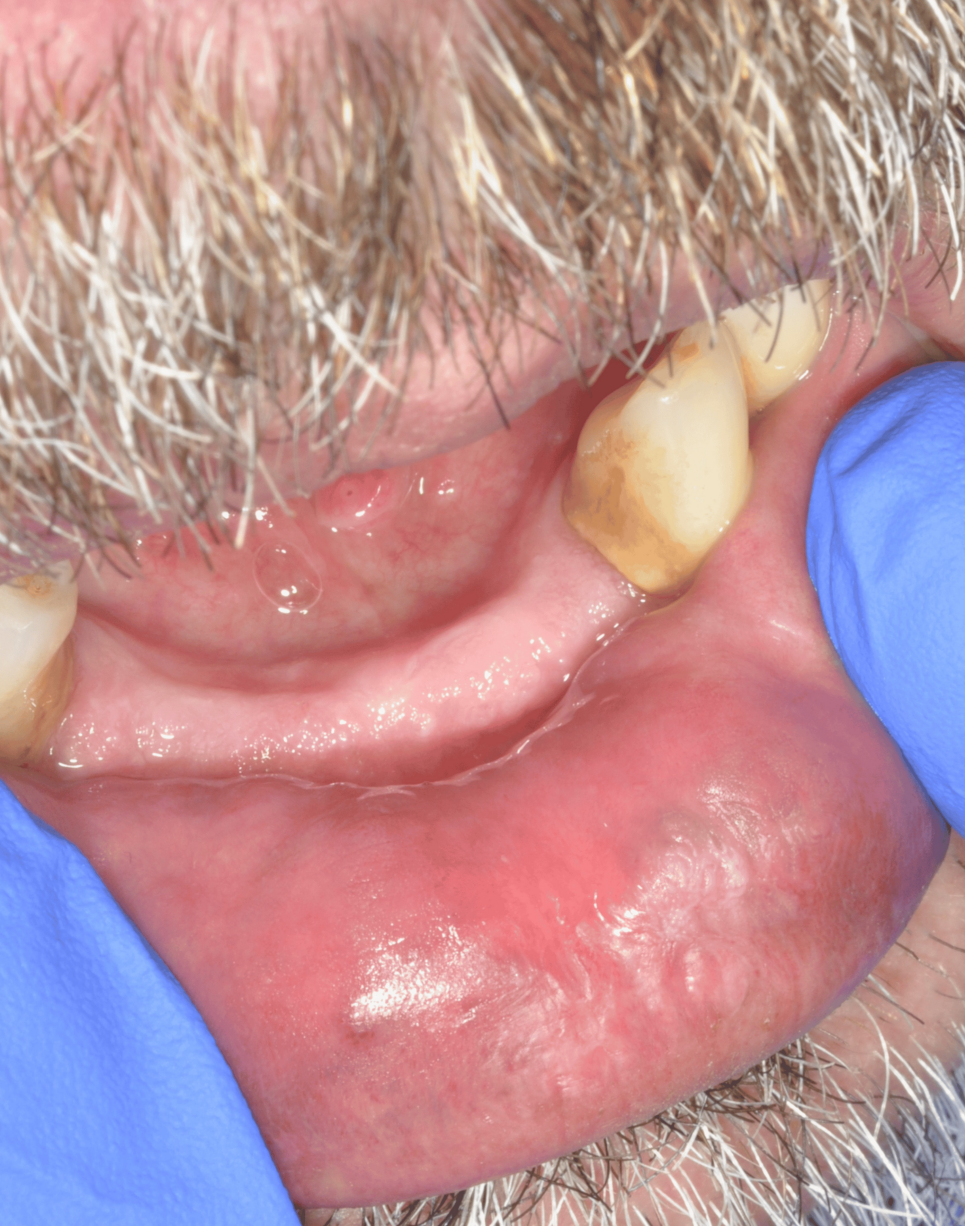
Two-year follow-up showing complete clinical remission of the leukoplakic lesions

At the two-year follow-up, the lesions had completely regressed without additional treatment. Clinically, the vermilion borders appeared healthy, with only a faint, flat, hypopigmented scar at the previous excision sites (Figure 7). No leukoplakic, verrucous, or indurated areas were observed, and no cervical lymphadenopathy was detected. Given the complete clinical resolution and absence of suspicious features, no repeat biopsy was deemed necessary. The overall clinical course was considered favorable under surveillance.

## Discussion

HPV, particularly the high-risk genotype HPV-16, plays a major role in the development of mucosal and cutaneous squamous cell carcinomas. HPV is primarily transmitted through direct contact, including sexual transmission, oro-genital contact, and, less commonly, autoinoculation through manual contact. Viral carcinogenesis mainly involves integration of the viral genome into the host cell DNA, leading to overexpression of the viral oncoproteins E6 and E7. These oncoproteins promote degradation of tumor suppressor proteins p53 and pRb, resulting in uncontrolled cell proliferation and genomic instability [6,11,12].

HPV is implicated in approximately 39% of vulvar carcinomas, 90% of anal carcinomas, and between 6% and 25% of head and neck squamous cell carcinomas, with a particularly high prevalence in oropharyngeal squamous cell carcinomas, where HPV positivity ranges from 52% to 71%. These cancers are predominantly associated with HPV-16 infection [6,10,11].

While the association between HPV-16 infection and squamous cell carcinoma of the upper aerodigestive tract, especially the oropharynx, is well established, the relationship between HPV infection and oral cavity squamous cell carcinoma remains controversial. This may be partly explained by differences in transmission routes, including oro-genital contact and possible autoinoculation, which may result in heterogeneous viral exposure within the oral cavity [5,10,12-16]. Several studies have reported highly variable HPV prevalence in oral cavity cancers, suggesting heterogeneity related to geographic factors, patient populations, detection methods, and anatomical subsites [17].

Sri et al. demonstrated a statistically significant association between HPV infection and oral squamous cell carcinoma in their cohort [5]. Conversely, the meta-analysis conducted by Katirachi et al., including more than 5,000 patients, found that only a limited number of studies reported a significant association between HPV and oral cavity squamous cell carcinoma, suggesting that HPV may play a less direct oncogenic role in this anatomical site compared with the oropharynx [10,14]. Recent data further support the hypothesis that HPV plays a dominant etiological role in oropharyngeal cancers, whereas its involvement in oral cavity carcinogenesis remains debated, potentially acting as a contributory rather than an exclusive causal factor [18-20].

The presence of leukoplakia associated with HPV-16 infection in the absence of histological dysplasia raises questions regarding the prognostic significance of isolated viral infection. Previous studies have shown that the risk of malignant transformation of oral leukoplakia primarily depends on the degree of epithelial dysplasia; however, HPV infection may influence the biological behavior of lesions, particularly by promoting persistence and recurrence after surgical treatment [2,18]. The early recurrence observed in our patient following surgical excision is consistent with this hypothesis and highlights the complexity of managing HPV-associated oral lesions.

From a clinical perspective, this case underlines the importance of a comprehensive diagnostic approach, including both oral and cutaneous examination. The concomitant presence of cutaneous verrucous lesions suggests a diffuse viral infection, reinforcing the need for multidisciplinary management involving oral surgeons, dermatologists, and otolaryngologists.

Surgical excision remains the treatment of choice for potentially malignant oral lesions in order to prevent malignant transformation [14]. Nevertheless, the risk of persistence or recurrence, particularly in the context of viral infection, has led to the development of alternative and adjunctive therapeutic approaches. These include ablative techniques such as laser ablation, cryotherapy, and electrocoagulation, which are indicated for localized lesions and allow targeted tissue destruction with preservation of surrounding structures. Photodynamic therapy (PDT) has also emerged as a minimally invasive option, particularly for superficial or multifocal lesions, with the advantage of repeatability and reduced morbidity. In addition, topical or systemic therapies have been explored, although their efficacy remains variable and evidence is still limited. Each modality presents specific advantages and limitations, and treatment selection should be individualized based on lesion characteristics and patient factors [15,16].

In the present case, these alternative modalities were not pursued due to the absence of dysplasia, the limited size of the lesions, and the favorable clinical evolution observed during follow-up. A conservative approach based on regular surveillance was therefore considered appropriate.

Furthermore, the concomitant presence of cutaneous verrucous lesions raises the possibility of viral autoinoculation, particularly through manual contact, as well as potential oro-genital transmission pathways. These mechanisms may contribute to viral persistence and multifocal involvement, and should be considered in the clinical evaluation and patient counseling.

Preventive strategies have also demonstrated efficacy, notably HPV vaccination, which is recommended for both girls and boys. Although its effectiveness in preventing HPV-related cancers is well established, its impact on oral cavity lesions remains to be fully clarified.

In conclusion, this case illustrates the complexity of the relationship between HPV-16 infection and leukoplakic lesions of the oral cavity. Tobacco use and alcohol consumption are well-established major risk factors for oral leukoplakia and oral squamous cell carcinoma, often acting as primary etiological drivers. In contrast, the role of HPV in oral cavity lesions remains less clearly defined and is considered to be contributory rather than causative in most cases. However, in the present case, the absence of tobacco and alcohol exposure reduces the likelihood of these confounding factors and supports a possible role of HPV in lesion development.

HPV has been detected in a variable proportion of oral leukoplakia and oral squamous cell carcinoma cases, with reported prevalence rates generally lower than those observed in oropharyngeal cancers. This suggests that, while HPV may contribute to lesion persistence or progression, it is unlikely to represent a dominant etiological factor in most oral cavity cases.

This case, therefore, highlights the importance of considering HPV infection in selected clinical contexts, particularly in patients without traditional risk factors. It emphasizes the need for accurate diagnosis and long-term clinical follow-up, even in the absence of histological dysplasia, to allow early detection of potential malignant transformation. This rare clinical presentation contributes to a better understanding of the role of HPV in potentially malignant oral disorders and highlights the need for further research in this field.

## Conclusions

HPV-16 infection is well recognized in the pathogenesis of oropharyngeal squamous cell carcinoma; however, its role in oral cavity potentially malignant disorders remains uncertain. This case highlights the diagnostic and therapeutic challenges associated with HPV-related leukoplakic lesions of the oral cavity.

An early clinical reappearance of whitish plaques at the same anatomical sites, with features similar to the initial lesions, was observed shortly after surgical excision, suggesting persistent epithelial alteration rather than true recurrence. This finding supports the hypothesis that persistent viral infection may influence lesion behavior and underscores the importance of careful clinical evaluation. Long-term surveillance is therefore essential in order to detect potential progression or malignant transformation at an early stage. Improved understanding of the clinical significance of HPV in oral leukoplakia may help guide future management strategies and optimize follow-up protocols for these uncommon presentations.
